# An Analysis of the Casting Polymer Mold Wear Manufactured Using PolyJet Method Based on the Measurement of the Surface Topography

**DOI:** 10.3390/polym12123029

**Published:** 2020-12-17

**Authors:** Paweł Turek, Grzegorz Budzik, Jarosław Sęp, Mariusz Oleksy, Jerzy Józwik, Łukasz Przeszłowski, Andrzej Paszkiewicz, Łukasz Kochmański, Damian Żelechowski

**Affiliations:** 1 Faculty of Mechanical Engineering and Aeronautics, Rzeszów University of Technology, 35-959 Rzeszów, Poland; gbudzik@prz.edu.pl (G.B.); jsztmiop@prz.edu.pl (J.S.); lprzeszl@prz.edu.pl (Ł.P.); l.kochmanski@prz.edu.pl (Ł.K.); 2 Faculty of Chemistry, Rzeszów University of Technology, 35-959 Rzeszów, Poland; molek@prz.edu.pl; 3 Faculty of Mechanical Engineering, Lublin University of Technology, 20-618 Lublin, Poland; j.jozwik@pollub.pl; 4 The Faculty of Electrical and Computer Engineering, Rzeszów University of Technology, 35-959 Rzeszów, Poland; andrzej.paszkiewicz@prz.edu.pl; 5 Prosolutions Majewscy Sp. J., 05-400 Otwock, Poland; damian.zelechowski@prosolutions.pl

**Keywords:** wax model, 3D printing, surface topography, stylus method, polymer mold, computer measurement system

## Abstract

An important factor having an impact on the condition of machine parts is their surface topography. For instance, in the production of a molded element in casting or injection molding processes, the surface topography of the molding cavity has a significant impact on the surface condition of the product. An analysis of the wear of a mold made with the PolyJet technique was performed in this work, and we examined the surface topography using the stylus method after casting a wax model of the turbine blade. The surface topographies showed a gradual degradation of the mold cavity surface. After the manufacture of 40 castings, there was a significant deformation of the microstructure of the mold cavity. The maximum height value (Sz) parameter had the most dynamic change from 18.980 to 27.920 μm. Its growth dynamics are mainly influenced by maximum peak height (Sp) rather than the maximum pit height (Sv) parameter. In the case of the root mean square height (Sq) and arithmetic mean height (Sa), their gradual increases can be seen from 2.578 to 3.599 μm and from 2.038 to 2.746 μm. In the case of the value of the skewness (Ssk) parameter, a small positive skew was observed. As for the kurtosis (Sku) values, the distributions are clearly leptokurtic.

## 1. Introduction

Currently, in the era of the rapid development of industry, manufacturing a physical model with complex geometry is not as difficult as it was a few years ago. The computer numerical controlled (CNC) machining, rapid prototyping (RP) and molding techniques have become indispensable branches of industry, allowing for almost any geometry to be manufactured [1,2,3,4]. They are used within the automotive [5,6], aviation [3,7] and medical industries [8,9]. Despite the significant development of RP methods fabricating models using metal alloys materials [10,11,12], the models are not always able to replace the details manufactured by traditional molding methods [13] (Table 1). This is particularly evident in the aviation industries in the case of heat-resistant nickel alloys. 

Currently, turbine blades are mainly made by precision vacuum casting [26,27]. Casting with controlled cooling allows obtaining blades with directed crystallization and monocrystalline blades. Such material structures are highly heat-resistant. Turbine blade surfaces are often cast by the investment method. In order to obtain internal cooling channels, special ceramic cores must be placed in the molds. For that reason, the investment casting process is still the basis of the mass production of gas turbine blades [28,29]. Investment casting is an industrial process base on lost wax casting [30,31,32,33,34]. In this process, the wax pattern is obtained by injection of wax into a master metal die mainly made from aluminum. The entire made wax assembly is then dipped in a ceramic slurry, covered with a sand stucco and allowed to dry. Once the ceramic has dried, the entire assembly is placed in a steam autoclave to remove most of the wax. The ceramic mold is then preheated to a specific temperature and filled with molten metal, creating the metal casting.

The casting patterns can be also made directly with 3D printing methods [35,36] from waxes or from other materials easily removable from the ceramic mold [4,37,38,39]. Taking into account industrial practice, such a solution does not always work well with the use of standard technological procedures. Some polymeric materials used in casting patterns can cause mold cracks when removed from the ceramic mold [40,41]. They are most often caused by the rapid emissions of gases caused by combustion of the polymeric model. Taking this into account, casting wax is still the best material for the production of casting patterns [31,32,35]. In addition, manufacturing a model of wax used normally in the production process does not require any technological changes or the working time of test equipment, but it can be done in a typical production line of a precision foundry. 

Some studies show the use of silicone molds for the production of wax casting patterns. That is a good solution taking into account the processing of casting wax, but it requires manufacturing additional tools, such as silicone molds [42,43,44]. It also takes a certain amount of time and entails additional costs. The longevity of this type of mold, considering the thermal impact of wax, is estimated to be for about 50 wax models. After this quantity of models, the degradation of the silicone mold cavity surface begins. 

An interesting alternative is the possibility of making a master mold from polymeric materials by 3D printing, intended for the production of a short series of casting models [45,46]. Various techniques are used in the process of direct mold production with the use of RP methods [4,47,48]. Recently, however, the PolyJet method has dominated the most [49,50,51]. This is due to the fact that using this method, high dimensionality and shape accuracy of the created models are obtained [52,53,54,55]. In the case of 3D printers working in the PolyJet system, it is possible to use polymeric materials with different properties; some of them are also transparent [56]. 

Wear is defined as the decrease in the performance of the work surface. Wear can be caused by many different physical and chemical processes during operation. The wear mechanisms are very complex, as they involve many interrelated factors, whose intensities depend on the type of working environment of machined parts, and on the types and the sizes of the operating parameters. An important factor having an impact on the condition of machine parts is their surface topography. During the machining process, the geometry of the surface topography changes. The surface impacts not only the wear of the mating parts but also the thermal processes during operation. Additionally, in the case of the production of molded elements in casting or injection processes, the surface topography of the molding cavity has a significant impact on the surface condition of the product [57,58]. During the process of surface topography inspection, optical and stylus measurement methods are used [59,60,61]. In the case of optical systems, laser triangulation [62,63], interference microscopy [64,65], confocal microscopy [66,67,68] and focus variation microscopy [69,70,71,72] are the most popular; however, the better repeatability results have been obtained using the stylus measurement method [61,73]. It is particularly visible when measuring the surface topography of an element made of a reflective material [61]. The stylus measurement method is based on the principle of mapping the surface by a blade that moves along the test surface at a certain speed [59]. When analyzing the literature related to the study of the surface topography of models produced using 3D printing, many research trends can be distinguished. The first group of publications refers to the analysis of the surface topography of research models manufactured with the use of various rapid prototyping methods [74,75,76,77]. In this respect, some authors analyze the influence of the position of the manufactured prototype in relation to the 3D printer’s space on the surface quality [78]. Some of the publications in the literature also relate to the analysis of surface topography of models manufactured by hybrid methods [79,80,81,82,83]. With hybrid methods, one obtains ready-made models in two ways:

- Indirectly, when the tool is manufactured on the basis of a pattern created with the use of RP;
- Directly, when intermediate steps are omitted and the model created with the RP serves as a lost model or as a tool forming a casting or molding model.

The major impact on investment casting is the ability to make high-quality patterns, but manufacturing the injection die is often costly and requires several months of lead time. Therefore, it is important to develop new solutions to improve the investment casting method. An interesting alternative is the possibility of making a master mold from polymeric materials by 3D printing, intended for the production of a short series of wax casting models. Currently, however, there are no studies that specifically take into account the wear of mold cavities made direct with the use of RP techniques. For this purpose, the manuscript presents an analysis of the wear of a mold made with the PolyJet technique after the production of 100 casting wax models, done by examining the surface topography after casting another 10 wax models of the turbine blade. In particular, the focus was on the evaluation of the parameters arithmetic mean height Sa, maximum height value (Sz), maximum peak height (Sp), root mean square height (Sq), maximum pit height (Sv) and those determining the skewness (Ssk) and kurtosis (Sku) of the obtained surface. The knowledge of the results of the mold wear should be the starting point for the development of a highly efficient procedure for the production of a short series of casting wax models, with the use of molds made with 3D printing techniques.

## 2. Materials and Methods 

**Figure 1:**
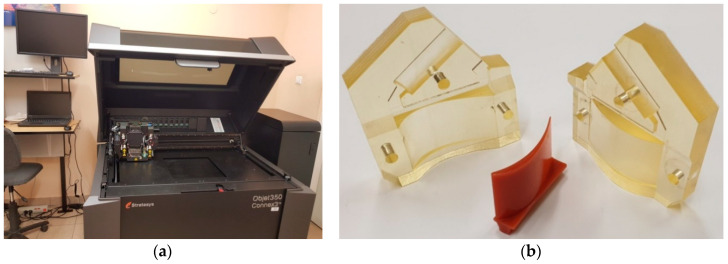
The process of 3D printing and wax castings; (**a**) Objet350 Connex 3 3D printer (**b**) the mold and the wax model.

The model of the tested form was designed in Computer Aided Three-dimensional Interactive Application (CATIA) software [84], and then saved in the Standard Triangle Language (STL) format during the tessellation process. In the next step, the file was loaded into the Objet Studio software [85]. A device was used in the process of manufacturing the mold Objet350 Connex 3 (Stratasys, MN, USA), which allows printing the model in PolyJet technology (Figure 1a). The RGD720 liquid photopolymer resin (Stratasys, MN, USA) was used in the printing process, and it guarantees high dimensionality and shapes stability of the created models. The thickness of a single layer in the printing process was 14 µm, and for the finishing style, gloss was used. With such selected parameters, it was possible to obtain a very smooth surface, which later allowed for easier demolding of the cast models. In the next step, the wax casting procedure of the blade was carried out using a form printed via PolyJet technology. The entire process took approximately 180 min. It consisted of several stages: heating up and taking the mold out of the furnace, pouring wax into the mold and cooling the mold. The mold was filled with KC 6052D casting wax, which was heated to a temperature of 100 °C, and then demoulded to obtain a wax model of the blade (Figure 1b). 

**Figure 2:**
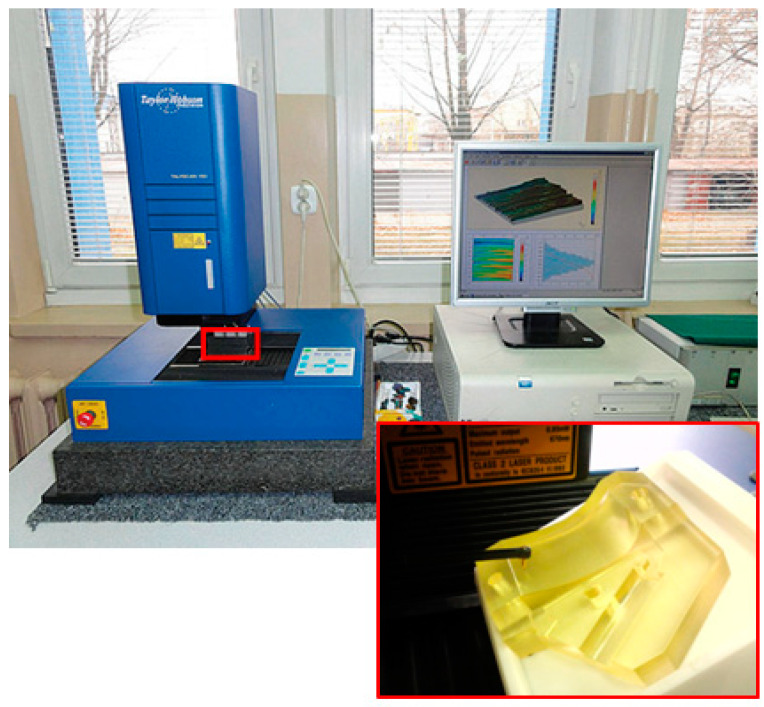
Measurement of the surface topography of the mold with the use of the stylus method.

The temperature change was measured with the use of an E Type Thermocouple (Nickel-Chromium/Constantan). Mold cavity wear was measured using a profilometer, Taylor Hobson TalyScan 150 [86,87], with a stylus rounding radius tip 2 μm. First, the surface topography of the PGN-3 standard was measured. The PGN-3 standard is characterized by a periodic surface structure, which is consistent with the surface structures of models obtained with RP methods. Basing on the standard selected, the accuracy and repeatability of the measurement have been verified. In order to obtain reliable measurement results, they were repeated 10 times on the standard. In the process of assessing surface topography, the standard was set to sampling steps along the *x* and *y* axes with minimum values of 5 μm. The single measured area had dimensions 3 mm × 3 mm. The lowest available measurement speed was used during the measurements of 2000 µm/s. Based on the analyzed surface, the average measuring range of the head was selected—392 µm/58,978 digits. During the measurement of one profile, the head was not raised before doing the next one. This procedure allowed for the avoidance of the introduction of unnecessary oscillations during the measurement. Then, in the same settings, the measurement of the mold cavity topography was done with the use of the PolyJet method. The surface of the mold is periodic—typical for elements manufactured with RP methods. Periodicity structure results from building the model layer by layer. The measurement of the mold cavity surface topography was carried out perpendicular to the direction of the printed layers (Figure 2).

## 3. Results

**Figure 3:**
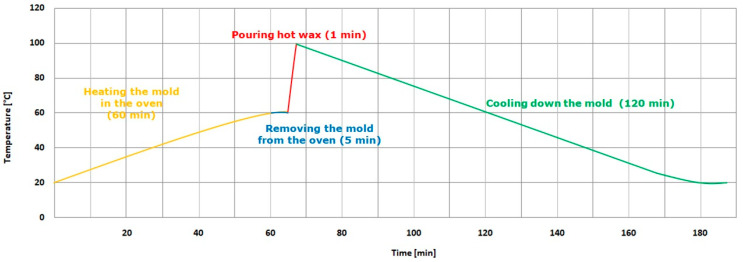
The temperature–time diagram of the casting of the turbine blade.

In the research, the temperature was measured first (Figure 3). For this purpose, we used an E Type Thermocouple (Nickel-Chromium/Constantan).

**Figure 4:**
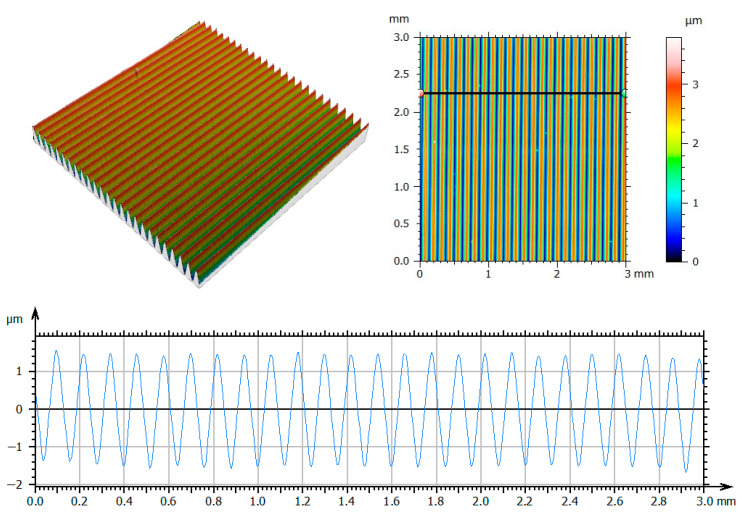
Obtained surface topography and selected profile for the first measurement of the standard.

First, the results of the repeatability of the stylus measurements were obtained within the PGN-3 standard’s framework. The data were analyzed in Mountains Map software [88]. In the process of determining the surface roughness, a filtration process was carried out, which firstly involved removing the obtained shape deviations. Then, in order to separate the long-wave components, a profile filter λc = 0.8 mm was used, which marks the transition from roughness to waviness. As a result, the surface roughness was obtained (Figure 4). Based on 10 measurements of surface of the standard, we the determined the profile Ra (arithmetical mean height of the profile) and Rz (maximum height of profile), and Sa (arithmetic mean height) and Sz (maximum height–parameters as shown in Table 1.

In order to analyze the statistical values, first the value of the arithmetic mean deviation was determined in accordance with Equation (1):

(1) $$\bar{y}=\frac{1}{n}\sum_{i=1}^{n}y_{i}$$

where: $\bar{y}$ arithmetic mean of the series of measurements; *y_i_*—next measurement results; *n*—number of measurements.

Then, the standard deviation for a single measurement result(s) was calculated, which is a measure of the dispersion of the experimental results around the mean value (Equation (2)):

(2) $$s=\sqrt{\frac{\sum_{i=1}^{n}{\left(y_{i}-\bar{y}\right)}^{2}}{n-1}}$$

and then came the standard deviation of the mean value (*S_r_*; Equation (3)):

(3) $$s_{r}=\frac{s}{\sqrt{n}}$$

In Table 2 statistical parameters obtained on the PGN-3 standard model are presented.

**Figure 5:**
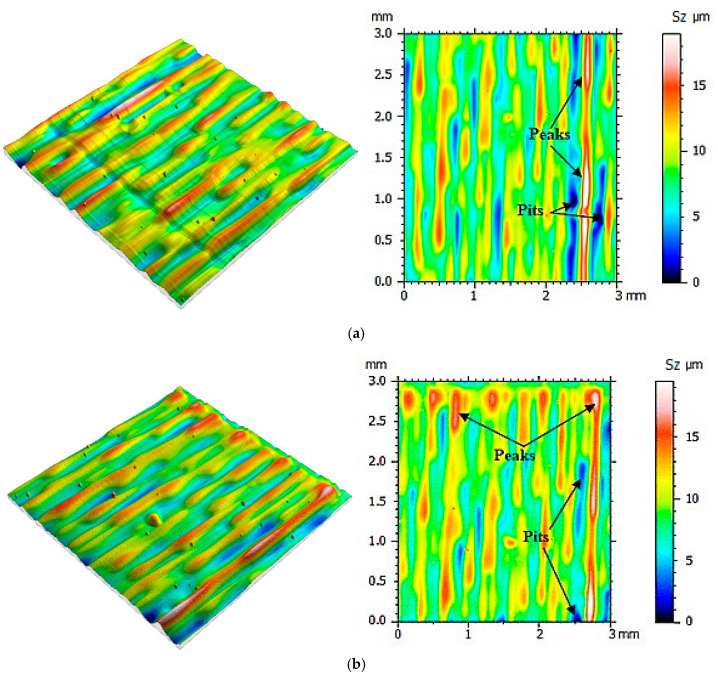
Visualization of the mold cavity surface topography: (**a**) before making the castings, (**b**) after making 10 castings.

**Figure 6:**
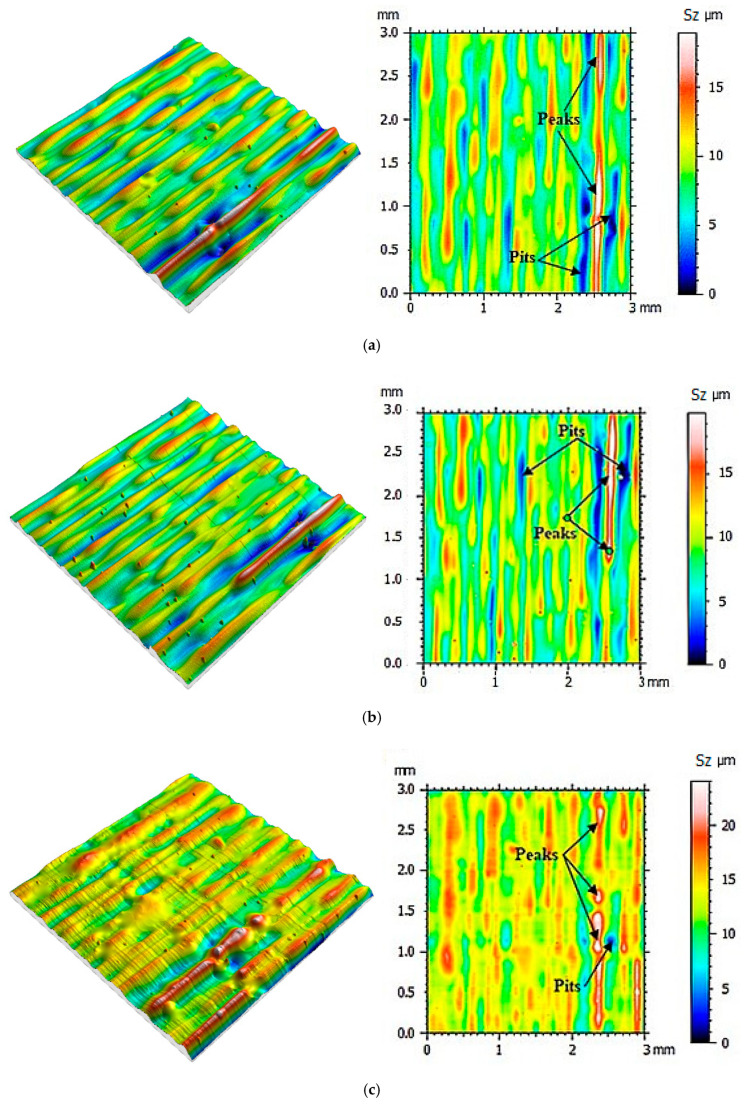
Visualization of the mold cavity surface topography: (**a**) after making 20 castings, (**b**) after making 30 castings, (**c**) after making 40 castings.

**Figure 7:**
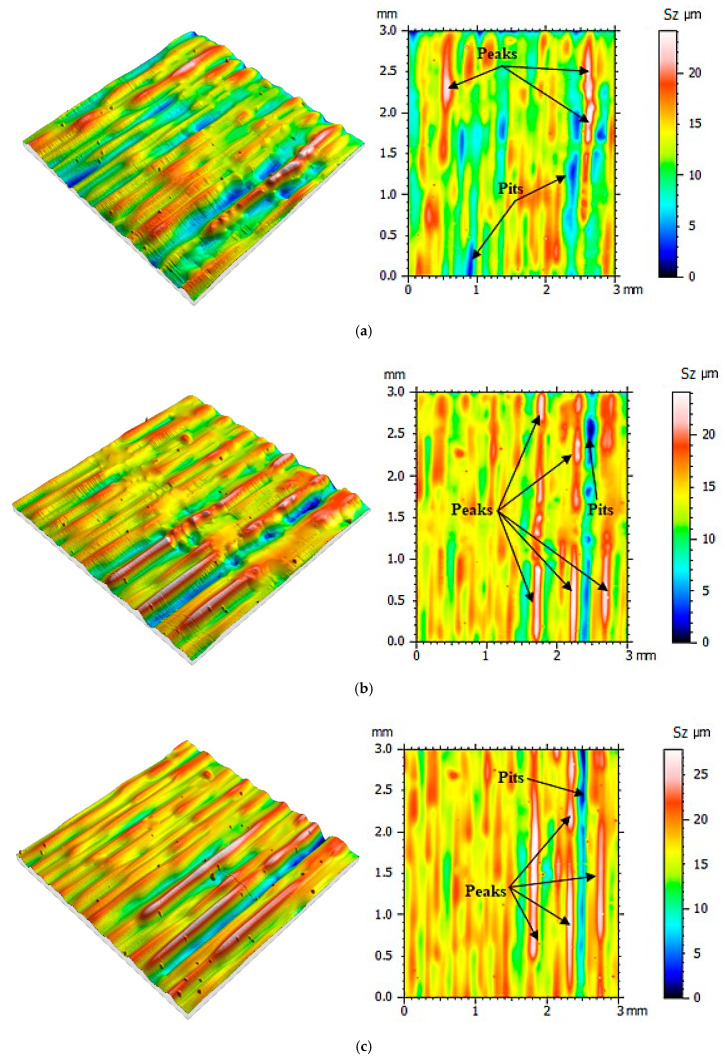
Visualization of the mold cavity surface topography: (**a**) after making 50 castings, (**b**) after making 60 castings, (**c**) after making 70 castings.

**Figure 8:**
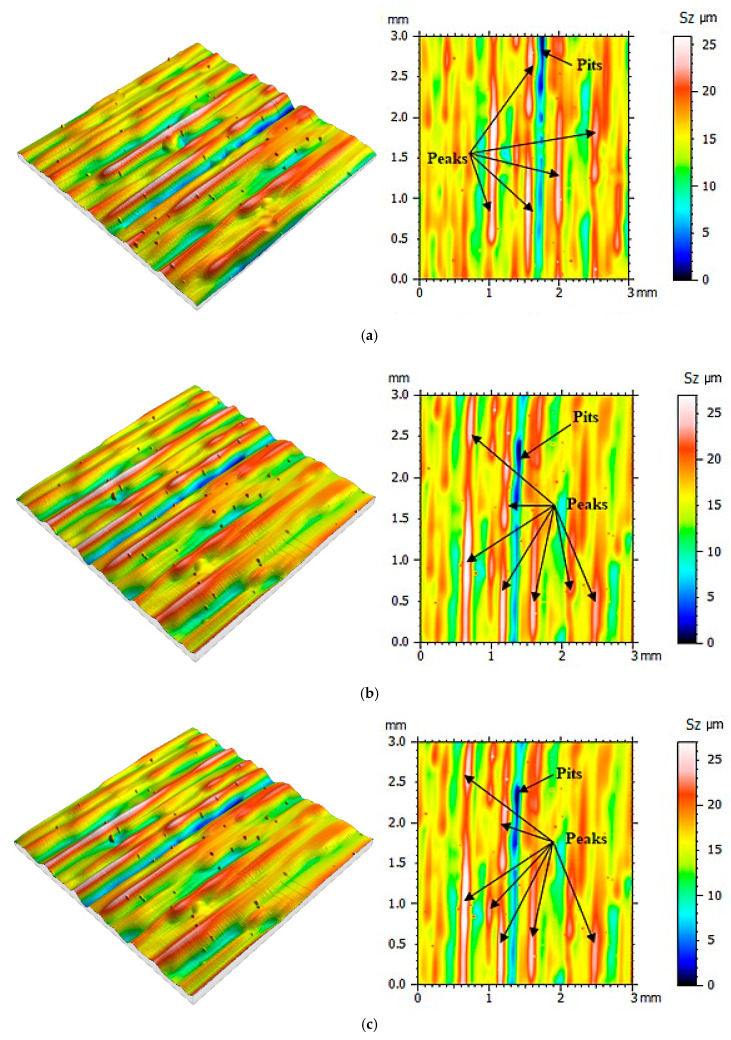
Visualization of the mold cavity surface topography: (**a**) after making 80 castings, (**b**) after making 90 castings, (**c**) after making 100 castings.

Then the same measurement protocol and data processing methods were used in the process of determining the mold cavity roughness topography made when using the PolyJet method. The surface roughness was determined after the next 10 casts were made, and until 100 castings were reached. The mold cavity surface topographies are presented in Figure 5, Figure 6, Figure 7 and Figure 8. 

As a result of discovering the surface topography of the mold cavity, values of the parameters *S_a_*, *S_z_*, *S_p_*, *S_q_*, *S_v_*, skewness (*S_sk_*) and kurtosis (*S_ku_*) were able to be calculated. *S_a_* parameter is the extension of Ra (arithmetical mean height) to a surface. It is the arithmetic mean of the absolute of the ordinate values within a definition area (*A*) (Equation (4)):

(4) $$S_{a}=\frac{1}{A}\iint_{A}\left|z\left(x,y\right)\right|dxdy$$

A root mean square (*S_q_*) value of the ordinate values within a definition area (*A*) is expressed as Equation (5):

(5) $$S_{a}=\frac{1}{A}\iint_{A}\left|z^{2}\left(x,y\right)\right|dxdy$$

*S_v_* parameter is defined as the smallest pit height value within a definition area, and *S_p_* parameter is the largest peak height value within a definition area. The *S_z_* parameter is the extension of Rz (maximum height of profile); it is expressed as the sum of the maximum peak height value and the maximum pit height value within a definition area (Equation (6)):

(6) $$S_{z}=S_{v}+S_{p}$$

The *S_sk_* parameter is the quotient of the mean cube value of the ordinate values and the cube of *S_q_* within a definition area (*A*) (Equation (7)):

(7) $$S_{sk}=\frac{1}{S_{q}^{3}}\left[\frac{1}{A}{\iint_{A}z}^{3}\left(x,y\right)dxdy\right]$$

The *S_ku_* parameter is the quotient of the mean quartic value of the ordinate values and the fourth power of *S_q_* within a definition area (*A*) (Equation (8)):

(8) $$S_{sk}=\frac{1}{S_{q}^{4}}\left[\frac{1}{A}{\iint_{A}z}^{4}\left(x,y\right)dxdy\right]$$

**Figure 9:**
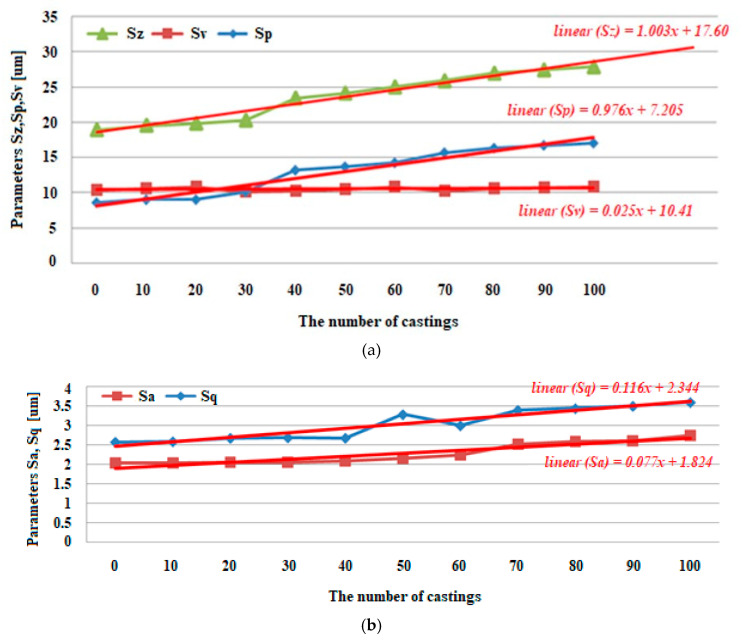
Diagrams representing the relations between; (**a**) *S_z_*, *S_p_* and *S_v_* parameters, and (**b**) *S_a_* and *S_q_* parameters depending on the number of castings.

In order to better visualize the amplitude parameters presented in Table 3, they are represented in Figure 9, taking into account the parameters’ values after different numbers of castings. The nature of the presented results indicates the possibility of adjusting a linear function with this data. For this purpose, linear regression analysis was used. In such a case, the regression line is selected so that the sum of the squared distances of all empirical points from the corresponding points of the regression line is as small as possible.

**Figure 10:**
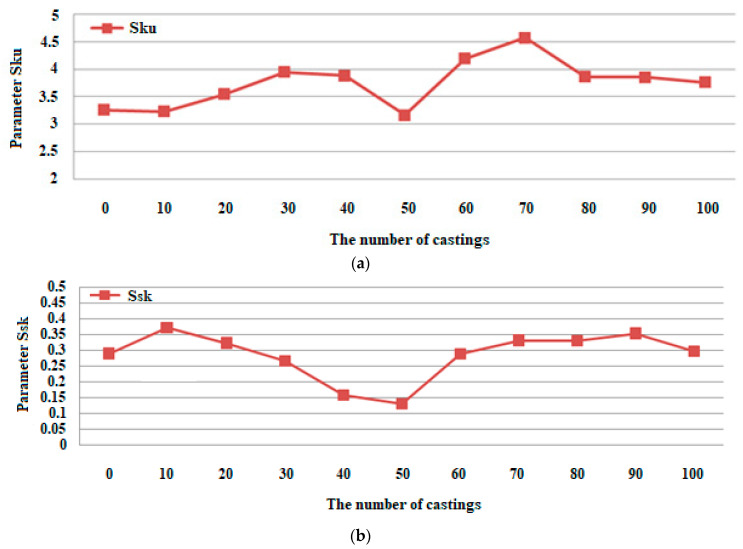
Diagrams representing the relations between; (**a**) kurtosis and (**b**) skewness, depending on the number of castings.

As an addition to the information of the mold cavity wear process on Figure 10, we present the changes of kurtosis and skewness parameters depending on the number of castings.

## 4. Discussion

In order to obtain reliable results, it was necessary at the research stage to carry out the process of assessing the repeatability of the measurements on the profilometer (3D Talyscan 150 Taylor Hobson, Leicester, UK). For this purpose, we chose the PGN-3 standard, which was characterized by a periodic surface structure, which was consistent with the surface structure of models obtained by 3D printing methods. On the basis of the comparison of the values of the standard parameters and the determined statistical parameters of Table 2, the entire procedure was developed not only for the measurements but also for digital data processing, which was then implemented during the assessment of the parameters of the mold cavity’s surface topography.

From the surface topographies presented in Figure 5, Figure 6, Figure 7 and Figure 8, a gradual degradation of the mold cavity surface is noticed. Despite heating the mold to 40 degrees Celsius in the oven, in order to avoid a violent reaction of the polymer structure to the temperature of the hot wax, after the manufacture of 40 castings, there was a significant deformation of the microstructure of the mold cavity, as shown in Figure 6c. At the analyzed moment, the surface of the mold cavity did not for many cracks. However, its periodic surface microstructure under the influence of the temperature of the poured wax changed significantly, which can be seen in Figure 7 and Figure 8. From the perspective of the topographies obtained before the manufacture of 40 castings, the periodicity of the structure can be seen quite accurately, with the observation of similar numbers of peaks and pits on the obtained surface (Figure 5 and Figure 6). In the case of the topographies presented in Figure 7 and Figure 8, an increase in the number of peaks above the pits can be seen. Despite the significant changes occurring between the 30th and 40th castings, no such rapid growth was observed in the subsequent series. Of course, the mold cavity topography was subject to further degradation, but less dynamic degradation. Regarding the values of the parameters from different numbers of castings (see Table 3), their change is close to linear. As a result of applying linear regression analysis, a linear function was fitted to the data. Based on the graphs presented in Figure 9 and Figure 10, it can be concluded that the value of the parameter Sz has the most dynamic change. Its growth dynamics are mainly influenced by Sp rather than the Sv parameter. This is due to significant differences in the obtained values of the slope for the fitted linear functions. For the Sp parameter it was 0.976 μm, and for Svit was 0.025 μm. Figure 9 also shows significant increases in Sp parameters, and thus Sz, after 40 castings. This is consistent with the graphic presentation of the surface topography in Figure 6c. In the case of the parameters Sq and Sa, which are presented in Figure 9b, their gradual increase can be seen, but it is not as dynamic as in the case of the parameters Sq and Sz. In the case of the value of the Ssk parameter, a small positive skew was observed. When analyzing the kurtosis values, the distributions can be seen to be leptokurtic.

## 5. Conclusions

The issue of research on the surface topography of mold cavities made with 3D printing techniques is currently not sufficiently presented. There is a lack of publications correlating the parameters of the surface topography with the degree of wear of the cavity mold. This is of particular importance for most technically applicable polymeric materials. The results presented in the manuscript are very important and provide information about the usefulness of using this type of solution in the future for the production of short series of wax casting models. On the basis of the obtained results, further research should be carried out on the selection of the casting method, casting material, the method of removing the model and the predicted accuracy of the prototype, in order to increase the efficiency of the manufacturing process of wax casting models using polymer molds.

## Figures and Tables

**Table 1:** The most common types of casting methods.

	Casting Method	Advantages	Disadvantages
**Expendable**—Mold is Made of Various Types of Binders Bonding Agents (Sand, Plaster, Ceramics)	**Sand casting**—characterized by using sand as the mold material [14,15]	Tooling cost are low; Relative easy process; Sand in most cases can be reused	Single use of mold; Poor surface finishing; Limited design freedom
	**Shell casting**—the mold is a thin shell of sand held together by resin binder [16,17,18]	Smoother cavity surface; Good dimensional accuracy; Machining often not required	Difficult justify for small quantities; More expensive metal pattern
	**Plaster casting**—similar to sand casting except that mold is made of plaster of Paris material [19]	Low tooling cost; Good dimensional accuracy and surface finishing	It can only be used with lower melting temperature;
	**Ceramic casting**—similar to plaster mold casting except that mold is made of ceramic material [20]	Complex shapes can be produced; High homogeneous slurries can be produced	Lower dimensional accuracy; Low production rate
	**Investment casting** **(Lost—wax)**—a mold is formed around a pattern of wax or similar material which is then removed by melting [21,22]	Good dimensional accuracy and surface finishing; Can be automated; Complex shapes with fine details can be made	Less strength than die cast parts; More steps are involved in production
**Permanent**—mold is made of metal	**Die casting**—molten metal is injected into mold cavity under high pressure [23,24]	Economical for large production; Good dimensional accuracy and surface finishing	Generally limited to metals; Part geometry must allow removal for die cavity
	**Centrifugal casting**—method of producing casting by pouring the molten metal into rapidly rotating mold [25]	Good surface finishing and accuracy; Low equipment cost; Can form very large parts	Limited to the cylindrical parts; Long lead time possible

**Table 2:** The statistical parameters obtained from the experimental data.

	Standard Value [μm]	Mean Deviation $\left(\bar{y}\right)$ [μm]	Standard Deviation (*s*) [μm]	Standard Deviation of the Mean Value (*S_r_*) [μm]
Ra	0.910	0.878	0.003	0.001
Rz	3.100	3.054	0.023	0.010
Sa	0.900	0.878	0.004	0.002
Sz	3.100	3.185	0.028	0.016

**Table 3:** Surface topography parameters.

	*S_q_* [μm]	*S_sk_*	*S_ku_*	*S_v_* [μm]	*S_p_* [μm]	*S_z_* [μm]	*S_a_* [μm]
Without casting	2.578	0,287	3.260	10.440	8.590	18.980	2.038
After 10	2.590	0.372	3.235	10.600	9.001	19.600	2.044
After 20	2.680	0.320	3.551	10.880	9.012	19.890	2.056
After 30	2.692	0.267	3.948	10.190	10.140	20.340	2.062
After 40	2.681	0.158	3.886	10.300	13.190	23.490	2.088
After 50	3.286	0.131	3.170	10.500	13.690	24.190	2.157
After 60	3.001	0.290	4.194	10.830	14.240	25.070	2.251
After 70	3.398	0.329	4.571	10.250	15.690	25.940	2.516
After 80	3.443	0.329	3.865	10.680	16.330	27.020	2.596
After 90	3.502	0.351	3.857	10.750	16.740	27.490	2.611
After 100	3.599	0.297	3.760	10.870	17.050	27.920	2.746
